# Life v2.0 - Quo vadis *Homo sapiens*?

**DOI:** 10.1186/1478-811X-8-9

**Published:** 2010-06-04

**Authors:** Stephan M Feller

**Affiliations:** 1 Cell Signalling Group, Department of Molecular Oncology, Weatherall Institute of Molecular Medicine, John Radcliffe Hospital, Headley Way, Oxford OX3 9DS, UK

## Abstract

This editorial briefly discusses the implications of the recent report by Craig Venter et al. on re-creating Mycoplasma mycoides as a synthetic life form.

## 

Some of our fellow human beings will surely see the 20th of May 2010 as the day of our second eviction from paradise, or even the first step on a sliding slope to hell. For others it may mark the day when hope for a new age of human prosperity emerged, similar to the day when Prometheus brought fire to the humans. Prometheus, of course, personally paid dearly for his action and we can only hope that history does not repeat itself in this aspect; there is no doubt in my mind that the 'infotainment' media will once again play a prominent role in hyping up this latest accomplishment, thereby generating unnecessary anxiety or even hate and a thoroughly unrealistic picture of the possible spin-offs.

So what has actually happened?

Craig Venter, a seemingly tireless maverick/maniac/visionary, and his team have succeeded in copying, i.e. re-producing, and slightly modifying an existing life form - something that all living cells have been able to do for a long time already. The difference is, of course, that *Homo sapiens *can now re-create, and - at least on the DNA level - at will modify, *Mycoplasma mycoides*. This is really quite an amazing accomplishment and it was over a decade in the making [1,2]. Amongst other things, this 're-booting' of a cell shows that - at least in this simple organism - the 'programmed software', i.e. the genetic blueprint, seems to be able to fully instruct and facilitate the complete replacement of the cellular 'hardware'.

Although this miniscule creature is potentially the most lowly version of all cellular life forms, its re-creation is clearly the beginning of many things. Not only is it the start of a new dimension in synthetic biology, it is probably also the real birthday of systems biology. So far, most so-called systems biologist have done at best 'sub-systems biology', but the new technologies developed by Venter et al. will enable completely unprecedented ways of systematic systemic experimentation with living cells. They should also make possible quite novel types of experiments for evolution biologists, signal transduction researchers and many others.

This is all good and well for the 'afficionados', but for the general public the real 'juice' is in the exciting new possibilities for practical applications, which are seemingly endless - if these technologies can also be applied to more complex genomes. A wide range of potentially useful, or harmful, but certainly patentable and commercially extremely interesting creatures may emerge.

Should we ever allow such human creations to live and work in the wild? Let them eat up oil spills or toxins? If not, this will limit their applications severely; and if we prohibit them to roam freely, what will happen if such creatures escape unintentionally or through a deranged individual? This is quite likely to happen, especially when commercialisation becomes a significant issue, which it probably will. Must all newly created life forms hence carry a fail-safe self-destruction device within them? If so, is it even possible to create a truly fail-safe system? Should the creation of new life forms be limited to microbes and other simple unicellular organisms? How do we prevent them from becoming pathogenic by horizontal gene transfer etc.? Do we want them on this planet, or should they be exiled, say onto the moon?

Of course, the synthetic organism currently at hand seems to be rather benign and, being a mutant version with over a dozen genes deleted, may at best give goats a mild mastitis, even if wilfully introduced into them. However, this is unlikely to be always true for future synthetic species, so the list of emerging questions is long, indicating that strict regulation and monitoring of such life v2.0 research activities will be required until we learn much more. Extensive debates about potential risks and applications by molecular biologists, ethics experts, legislators and the general public are clearly warranted which go beyond the commendable initial steps already made in this context [3,4]. Globally effective laws need to be developed and implemented within the next decade.

In a nutshell, life on this planet has once again become just a little more complex.
